# CXCR4/CXCL12 Signaling and Protumor Macrophages in Primary Tumors and Sentinel Lymph Nodes Are Involved in Luminal B Breast Cancer Progression

**DOI:** 10.1155/2018/5018671

**Published:** 2018-04-16

**Authors:** Carlotta Raschioni, Giulia Bottai, Andrea Sagona, Valentina Errico, Alberto Testori, Wolfgang Gatzemeier, Fabio Corsi, Corrado Tinterri, Massimo Roncalli, Libero Santarpia, Luca Di Tommaso

**Affiliations:** ^1^Oncology Experimental Therapeutics, Humanitas Clinical and Research Center, Rozzano, Milan, Italy; ^2^Senology Unit, Humanitas Clinical and Research Center, Rozzano, Milan, Italy; ^3^Thoracic Surgery Unit, Humanitas Clinical and Research Center, Rozzano, Milan, Italy; ^4^Laboratory of Nanomedicine, Surgery Division, Department of Biomedical and Clinical Sciences, University of Milan, “Luigi Sacco” Hospital, Milan, Italy; ^5^Breast Unit, Surgery Division, ICS Maugeri, Pavia, Italy; ^6^Pathology Unit, Humanitas Clinical and Research Center, Rozzano, Milan, Italy; ^7^Department of Biomedical Sciences, Humanitas University, Rozzano, Milan, Italy

## Abstract

Luminal B breast cancers (BC) have a more aggressive behavior associated with a higher rate of tumor relapse and worse prognosis compared to luminal A tumors. In this study, we evaluated the involvement of specific epithelial-to-mesenchymal transition- (EMT-) and immune-related pathways in the dissemination of luminal B BC cells. The expression of 42 EMT- and immune-related genes was evaluated in matched sentinel lymph nodes (SLNs) analyzed by the one-step nucleic acid amplification assay (OSNA) and primary tumors of 40 luminal B BC patients by gene array and immunohistochemistry. The results were validated in an independent group of 150 luminal B tumors by immunohistochemistry and immunofluorescence and using gene expression data from 315 luminal B BC patients included in the Metabric dataset. We found that the expression of *CXCR4* (*p* = 3.28*E* − 02) and *CD163* (*p* = 6.92*E* − 03) was significantly upregulated in SLNs of recurrent luminal B BC patients. Luminal B primary tumors overexpressing CXCR4 were characterized by an increased expression of vimentin and a high content of CD163-positive macrophages. Bioinformatics analysis confirmed the correlation of *CXCR4* with *CXCL12*, *VIM*, and *CD163* expression and LN involvement. Our results suggest that the upregulation of the CXCR4/CXCL12 pathway and the presence of protumor macrophages in the primary tumor and SLNs sustain the aggressiveness of an important subgroup of luminal B BC.

## 1. Introduction

Breast cancer (BC) is a heterogeneous disease, which encompasses distinct subtypes that differ in molecular features, clinical behavior, and response to treatment [1–3]. Gene expression-based classification identified four major BC molecular subtypes defined as luminal A and B, human epidermal growth factor receptor 2- (HER2-) enriched, and triple negative (TN)/basal-like tumors [1–3]. Luminal B tumors, which account for approximately 20% of all BC, show a lower expression of estrogen receptor (ER), lower or no expression of progesterone receptor (PgR), and higher proliferation compared to luminal A cancers and can be HER2 positive [4, 5]. In particular, luminal B tumors have a more aggressive behavior compared to luminal A cancers, showing a pattern of tumor recurrence and prognosis similar to those of HER2-enriched and TN/basal-like cancers [6].

It is well established that cancer cells, moving from primary breast tumor, can reach distant organs and metastasize through both blood and lymphatic vessels [7]. The sentinel lymph node (SLN), being by definition the first lymph node reached by BC cells spreading from the primary site, exerts a pivotal role in disease progression [8]. Tumor dissemination through SLNs, which can be rapidly detected through the one-step nucleic acid amplification assay (OSNA), is often driven by the epithelial-to-mesenchymal transition (EMT) process that allows epithelial cells to detach from the surrounding tissue and acquire a mesenchymal phenotype, gaining migratory and invasive abilities [9, 10].

In the last few years, a growing interest has been given to the relationship between tumor cells and the immune system [11, 12]. Importantly, EMT is emerging as a crucial mechanism regulating the dynamic interactions in the tumor microenvironment and supporting tumor immune escape [13, 14]. Indeed, cancer cells with mesenchymal features are able to shape the phenotype and the activity of tumor-associated immune cells, which in turn can regulate EMT in cancer cells through the release of multiple soluble mediators [13, 14]. In particular, inflammatory cells and tumor-associated macrophages (TAMs) have been shown to be able of inducing EMT, sustaining tumor progression in BC [13, 15]. Thus, the identification of the mechanisms underlying the acquisition of metastasis-enabling features and the generation of a permissive microenvironment for tumor growth and invasion can help identify luminal B BC patients at high risk of relapse and may represent the rationale for the development of novel therapeutic strategies.

In this study, we evaluated the role of EMT- and immune-related pathways in sustaining the dissemination to SLNs and in driving local and distant relapse in luminal B BC patients.

## 2. Methods

### 2.1. One-Step Nucleic Acid Amplification (OSNA)

The OSNA assay was performed as previously reported, using the OSNA BC System (Sysmex, Kobe, Japan)[16]. Briefly, after removing extranodal and fatty tissues, the SLNs (≤600 mg) were homogenized in 4 mL of Lynorhag lysis buffer (Sysmex) for 90 sec on ice using a Physicotron Warring blender with an NS-4 shaft (MicroTec Nichion) and then centrifuged at 10,000 ×g for 1 min at room temperature. SLNs exceeding the specified maximum weight of 600 mg were cut into two or more pieces and processed separately. The lysate (2 *μ*L) was subjected to the automated reverse transcription loop-mediated isothermal amplification (RT-LAMP) of cytokeratin 19 (CK19) mRNA using an RD-100i analyzer (Sysmex). The remaining sample was stored at −80°C. CK19 mRNA copy number was determined based on a standard curve generated using a known quantity of human CK19 mRNA, and samples were classified based on the CK19 mRNA copy number/*μ*L. Accordingly, we defined macrometastasis (++) as >5 × 10^3^ copies/*μ*L of CK19 mRNA, micrometastasis (+) as 2.5 × 10^2^ to 5 × 10^3^ copies/*μ*L, and nonmetastasis (−) as <2.5 × 10^2^ copies/*μ*L. All surgical resection specimens were also analyzed for CK19 expression using immunohistochemistry (RCK108 antibody, Dako).

### 2.2. Clinical and Pathological Data of Luminal B HER2-Negative Breast Cancer

We used a discovery cohort of 40 patients with invasive ductal luminal B (HER2-negative) BC and OSNA ++ SLNs, who underwent surgery at the Humanitas Clinical and Research Center between 2011 and 2014. The validation dataset included 150 patients with invasive ductal luminal B (HER2-negative) BC and OSNA ++ SLNs, who underwent surgery at the Humanitas Clinical and Research Center between 2006 and 2010. Samples in the two cohorts were homogeneous for all clinicopathological features and treatments. Matched formalin-fixed and paraffin-embedded (FFPE) tissues were available for all the selected cases. Patients were classified as relapsing based on the first evidence of invasive relapse at any site. Clinical and pathological data of these cohorts are reported in Supplementary Table 1 and Supplementary Table 2.

### 2.3. Sample Processing and Quantitative Reverse Transcription PCR (qRT-PCR)

The OSNA lysates were incubated at 37°C in a water bath until completely thawed and centrifuged at 12,000 ×g for 5 min at room temperature. The supernatant was discarded, and lysis reagent (Qiazol, Qiagen) was added. RNA was then extracted with the RNeasy Plus Universal Tissue Kit (Qiagen) following the manufacturer's instructions.

RNA retrotranscription was performed with the iScript Advanced cDNA Synthesis Kit (Bio-Rad). Briefly, the reverse transcription master mix was prepared as indicated, and 5 *μ*L of total RNA input were added to 15 *μ*L of the prepared master mix for each reverse transcription reaction. All the reactions were assembled on ice. The complete reaction mix was then incubated in a thermal cycler according to the manufacturer's protocol.

Based on a comprehensive literature review and pathway enrichment analysis, we identified a set of 42 genes associated with immune and EMT functions (Supplementary Table 3). Expression profiling by qRT-PCR was performed using a custom gene panel (Bio-Rad) using a ViiA 7 Real-Time PCR system (Applied Biosystems). Data were then normalized, and we considered as positive cut-off a ΔCt > 4.

### 2.4. Immunohistochemistry and Immunofluorescence

FFPE tissues were cut into 2 *μ*m sections and subjected to antigen unmasking at 98°C in a water bath for 25 min. Slides were cooled for 30 min before the treatment with Peroxidase Blocking Reagent (Dako) and Background Sniper (Biocare). Immunohistochemical reactions were performed using CONFIRM anti-Ki67 (clone 30-9; Ventana Medical Systems), CONFIRM anti-vimentin (clone V9; Ventana Medical Systems), anti-CD163 (clone 10D6, 1 : 1000; Novocastra), anti-CXCR4 (clone UMB2, 1 : 200; Abcam), and anti-CXCL12 (clone 79018, 1 : 500; R&D Systems) primary antibodies. DAB chromogen kit (Biocare Medical) was used for immunodetection. All analyses were performed centrally at the Humanitas Clinical and Research Center by two pathologists, who were blinded for patient characteristics and outcome. The staining was evaluated by intensity (0 = no staining, 1 = weak, 2 = moderate, and 3 = strong) and the percentage of stained cells (0 = 0%, 1 = 1–10%, 2 = 11–50%, 3 = 51–80%, and 4 > 80%). The immunoreactive score (IRS) was obtained by multiplying the intensity with the proportion of positive cells, and samples were classified as negative (IRS = 0–5) or positive (IRS = 6–12) [17–19]. CD163 staining was scored using a four-tiered system ranging from 0 (absent) to 3 (dense), as previously described [15].

For immunofluorescence, FFPE sections (3 *μ*m) were subjected to UV radiation for 48 h at 16°C. Deparaffinization and antigen unmasking were performed in sodium citrate buffer pH 6.0 (Bio-Optica) at 98°C in a water bath for 25 min. Slides were cooled in deionized water and blocked with PBS containing 2% bovine serum albumin and 2% goat serum for 30 min at room temperature. Sections were then incubated with anti-CXCR4 antibody (clone UMB2, 1 : 200; Abcam) for 1 h at room temperature and then with anti-CD163 antibody (clone 10D6, 1 : 1000; Novocastra) for 1 h at room temperature. Goat anti-rabbit Alexa Fluor 488 and goat anti-mouse Alexa Fluor 594 (Invitrogen) were used as secondary antibodies. Slides were counterstained with DAPI and mounted with ProLong Gold (Invitrogen). Images were captured using an Olympus Fluoview FV1000 laser scanning confocal microscope (Olympus).

### 2.5. In Silico Data Analysis

Gene expression data from the Metabric dataset were processed as previously described [20, 21]. Overall, we analyzed 315 patients with luminal B (HER2-negative) BC, as defined by the PAM50 classifier and based on the expression of the *ERBB2* gene. Patients' characteristics are reported in Supplementary Table 4.

### 2.6. Statistical Analysis

The analysis of differential gene expression between patients' subgroups was performed by unpaired two-tailed *t*-test. Spearman's rank correlation test was used to evaluate the correlation between variables. Clinicopathological associations were investigated using Fisher's exact test (FET) and Mann–Whitney *U* test for categorical and continuous data, respectively. For categorical analysis, the median expression of *CXCR4* was used as cut-off. *p* values were adjusted for multiple testing by Benjamini-Hochberg correction, and the level of statistical significance was set at *p* < 0.05.

## 3. Results

### 3.1. Identification of Genes Differentially Expressed between Relapsing and Nonrelapsing Luminal B BC Patients

We first investigated the potential involvement of EMT and immune signaling in the metastatic dissemination of luminal B BC. We analyzed the expression of 42 genes associated with these pathways in 40 OSNA ++ SLNs of luminal B (HER2-negative) BC patients, which included 20 relapsing subjects and 20 tumor recurrence-free patients (Supplementary Table 1).

We identified 12 genes that were differentially expressed between recurrent and nonrecurrent luminal B BC patients (Table 1). However, only eight genes (*CALD1*, *CD163*, *COL1A2*, *CXCR4*, *ITGAV*, *SNAI2*, *TGFB2*, and *TWIST1*), whose expression was upregulated in SLNs of patients who experienced tumor relapse, remained significant after adjustment for multiple testing (Table 1), indicating that both EMT and immune response have a crucial role in the spread of luminal B BC cells to SLNs. In particular, the chemokine receptor *CXCR4*, which mediates both the EMT process and the polarization toward an immunosuppressive microenvironment, has been directly involved in the progression of ER-positive BC [22–24]. Furthermore, the high expression of *CD163* in SLNs from relapsing patients may suggest an active role of M2 protumor macrophages in supporting the aggressiveness of luminal B BC cells.

### 3.2. CXCR4/CXCL12 Axis Has a Key Role in Determining the Metastatic Destination of Luminal B BC

**Figure 1:**
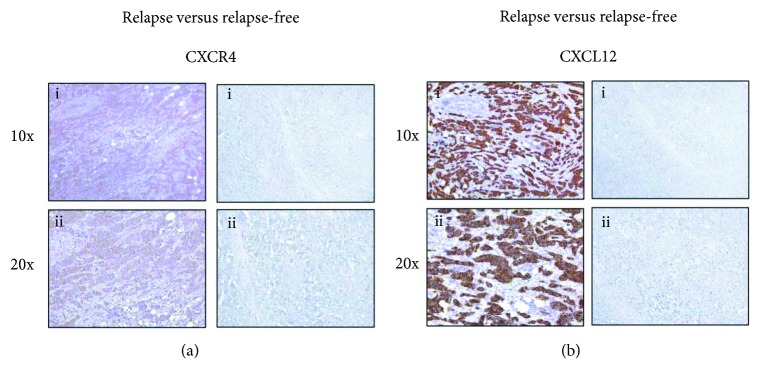
CXCR4 and CXCL12 expression in luminal B BC samples. (a) Immunohistochemical analysis showing a higher expression of CXCR4 protein in the primary tumors of relapsing compared to relapse-free luminal B BC patients. (b) Immunohistochemical analysis of CXCL12 protein levels in the primary tumors of relapsing and relapse-free luminal B BC patients. (i) 10x magnification and (ii) 20x magnification.

**Figure 2:**
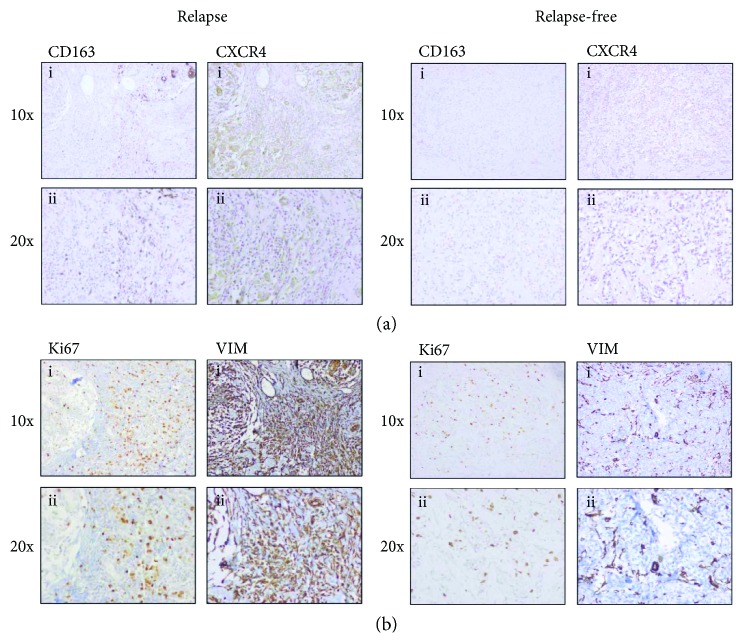
Immunohistochemical staining for CXCR4, vimentin, and CD163-positive macrophages in luminal B BC samples. (a) Immunohistochemical analysis of CXCR4 protein levels and the presence of CD163-positive macrophages in the primary tumors of relapsing and relapse-free luminal B BC patients. (b) Evaluation of Ki67 and vimentin protein expression in the primary tumors of relapsing and relapse-free luminal B BC patients. (i) 10x magnification and (ii) 20x magnification.

To evaluate the potential involvement of CXCR4- and CD163-positive macrophages in the progression of luminal B BC, we analyzed the matched primary tumors of luminal B BC patients in the discovery cohort. We found that CXCR4 protein was overexpressed in relapsing luminal B BC patients compared with nonrelapsing cases (FET *p* = 0.010; Figure 1(a)). Furthermore, we showed that primary tumors with significant high levels of the CXCR4 receptor also overexpressed its ligand CXCL12 (Figure 1(b)). The association between CXCR4 protein levels and tumor relapse (FET *p* = 0.031) was confirmed in luminal B BC patients of the validation cohort (FET *p* = 0.031; Figure 2). Importantly, we demonstrated that luminal B primary tumors overexpressing CXCR4 were characterized by an increased expression of the mesenchymal marker vimentin and by a high content of CD163-positive TAMs (FET *p* = 0.033; Figure 2). Conversely, we were not able to detect any association between the expression of CXCR4 and Ki67 in luminal B BC (Figure 2).

**Figure 3:**
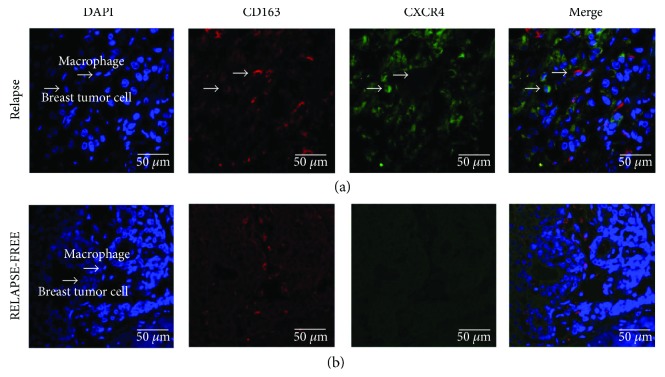
Double immunofluorescent staining for CXCR4 and CD163 in luminal B BC samples. Representative images of double immunofluorescent staining and confocal microscopy on primary tumors of relapsing (a) and nonrelapsing (b) luminal B BC patients showing that CXCR4-expressing cancer cells (green) and CD163-positive TAMs (red) are localized in the same tumor regions of relapsing luminal B BC. Scale bars represent 50 *μ*m.

To confirm the relevance of TAMs in favoring the aggressiveness of BC cells, we performed double immunofluorescence for CXCR4 and CD163 on tumor tissues of luminal B BC patients from the validation cohort. Accordingly, we demonstrated that CXCR4-positive BC cells and CD163-positive TAMs were localized in the same tumor regions in 75% of relapsing cases, while no association was observed in nonrelapsing luminal B cancers (Figure 3).

**Figure 4:**
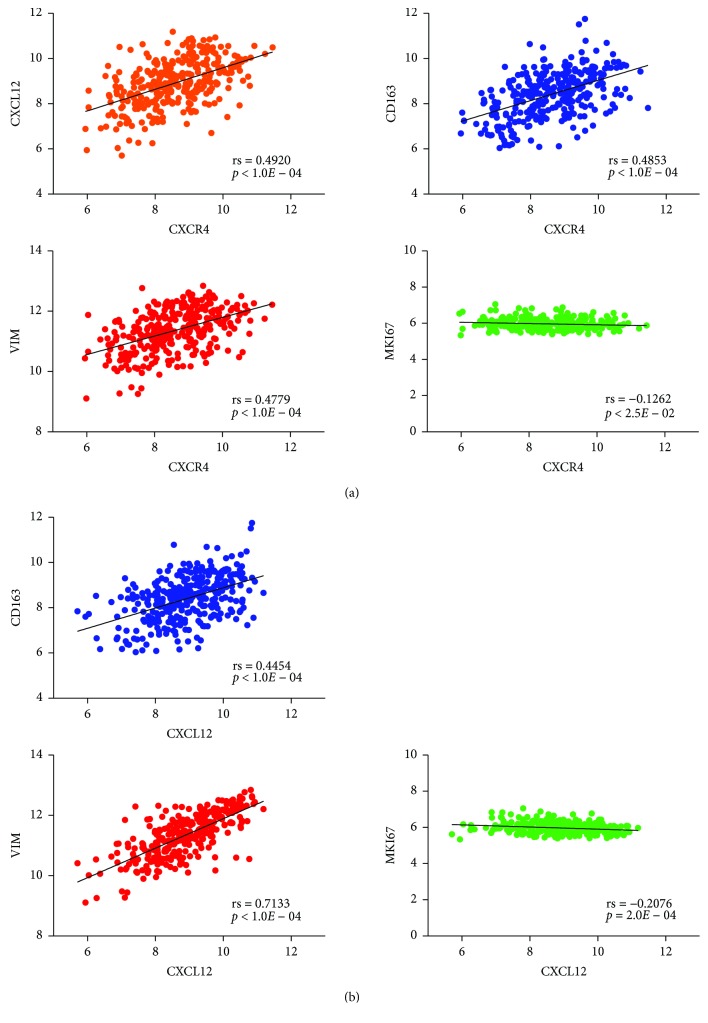
In silico analysis of gene expression data of luminal B BC patients from the Metabric dataset. (a) Correlation between *CXCR4*, *CXCL12*, *VIM*, *CD163*, and *MKI67* in 315 luminal B (HER2-negative) primary BC. (b) Correlation between *CXCL12*, *VIM*, *CD163*, and *MKI67* in 315 luminal B (HER2-negative) primary BC. Spearman's coefficients (rs) and *p* values are shown.

These findings were further validated by analyzing the gene expression profiles of 315 luminal B (HER2-negative) BC patients enclosed in the Metabric dataset. In silico analysis confirmed that the expression of *CXCR4* in the primary tumor significantly correlated not only with the expression of its ligand *CXCL12* (Spearman's coefficient, rs = 0.4920) but also with the expression of *VIM* (rs = 0.4779) and *CD163* (rs = 0.4853) (Figure 4(a)). Similarly, the expression of *CXCL12* was strongly associated with the expression of *VIM* (rs = 0.7133) and *CD163* (rs =0.4454) (Figure 4(b)). Conversely, we found a mild inverse correlation of *CXCR4* and *CXCL12* with *MKI67* (rs = −0.1262; rs = −0.2076, resp.), thus confirming the lack of association between the CXCR4/CXCL12 axis and proliferation in luminal B BC (Figures 4(a) and 4(b)). Furthermore, we demonstrated that the overexpression of *CXCR4* in luminal B (HER2-negative) BC was significantly associated with LN positivity according to both Mann–Whitney (*p* = 0.003) and FET tests (*p* = 0.001).

On overall, these results suggest that a permissive tumor microenvironment in the primary tumor and SLNs, characterized by EMT features such as the activation of CXCR4/CXCL12 axis and the presence of protumor M2 TAMs, can sustain the aggressiveness of cancer cells and support their metastatic dissemination in luminal B BC.

## 4. Discussion

Luminal cancers, which enclose around two-thirds of all BC, are generally considered less aggressive and associated with better prognosis compared with nonluminal tumors. However, luminal B cancers are recognized as having a metastatic dissemination time pattern and an outcome similar to those of HER2-positive and TNBC, with an increased risk of tumor relapse in the first five years after diagnosis [6]. SLN biopsy has been demonstrated to be an accurate predictor of axillary LN status, which is considered a consistent prognostic factor in BC including luminal B tumors and it is currently used in treatment decision-making [25–28].

Migration of primary tumor cells in the local lymphatic system facilitates the dissemination to the SLN and subsequently to distant organs. EMT and immunosuppression are increasingly recognized as potent inducers of tumor progression and invasion, although the mechanisms mediating the tumor spread to the regional draining LN are still not fully elucidated [9–15]. In this study, we demonstrated that the CXCR4/CXCL12 pathway was upregulated in the primary tumor and in the matched macrometastatic SLNs of luminal B BC patients who had tumor recurrence, and it was associated with characteristics of tumor aggressiveness and invasiveness such as LN involvement. Increased levels of CXCR4/CXCL12 have been found in many types of cancer, including BC, and have been associated with metastasis and poor prognosis [22, 29–32]. Accordingly, a strong expression of CXCR4 has been demonstrated in primary breast tumors, axillary LN metastases, and distant metastases of the lung and liver [30]. Interestingly, we found that the expression of CXCR4 correlated with the levels of its ligand CXCL12 in the primary tumors of relapsing luminal B BC patients. In agreement with previous findings showing that CXCL12 is preferentially expressed in the most common metastatic sites of BC (i.e., the LNs, lung, liver, and bone marrow) and that it induces the recruiting of CXCR4-positive cancer cells to CXCL12-expressing sites, our results suggest that the chemokine CXCL12 and its cognate receptor CXCR4 exert a key role in determining the metastatic potential of breast tumor cells [30, 33].

In ER-positive BC, the activation of CXCR4 signaling has been demonstrated to drive BC cells to an invasive and endocrine therapy-resistant phenotype through the activation of extracellular signal-regulated kinases (ERK) 1/2, p38 mitogen-activated protein kinase (MAPK), and NF-*κ*B pathways and through the enhancement of ER-mediated gene expression [23, 24, 34]. Furthermore, CXCR4/CXCL12 axis has been shown to be capable of inducing EMT in ER-positive BC through the regulation of key EMT markers such as E-cadherin and N-cadherin [23, 24]. Accordingly, we demonstrated that the CXCR4 pathway correlated with the expression of the mesenchymal marker vimentin in luminal B primary tumors and that the overexpression of *CXCR4* in metastatic SLNs was concurrent with that of other EMT-related genes, such as *SNAI2*, *TGFB2*, and *TWIST1*. Thus, our results provide an important evidence for the association of CXCR4/CXCL12 axis with the progression of tumor cells toward a mesenchymal phenotype, suggesting a potential mechanism that drives the metastatic spread of luminal B BC cells from primary tumors and SLNs to distant sites.

Growing evidence indicates that the metastatic ability of a cancer relies on intrinsic properties of tumor cells such as EMT and on signals derived from the tumor microenvironment [35]. Interestingly, numerous chemokines and associated receptors, including CXCL12 and CXCR4, have been shown to exert a key role in mediating the communication between cancer cells and nonmalignant stromal cells, ultimately favoring the establishment of a permissive microenvironment for tumor development and progression [22, 30, 36]. Accordingly, we found that *CD163* was overexpressed in metastatic SLNs of relapsing luminal B BC patients. Furthermore, we demonstrated that aggressive luminal B primary tumors characterized by the high expression of CXCR4 and CXCL12 showed an increased content of CD163-positive macrophages. Additionally, we revealed that protumor TAMs and CXCR4-positive cancer cells were frequently localized in the same areas of the primary tumor. Of note, TAMs, which essentially resemble alternatively activated M2-like macrophages, have been shown to affect multiple aspects of cancer progression, including EMT and immune suppression, and to modulate the response to anticancer therapies [15, 37, 38]. Moreover, mononuclear phagocytes are attracted to tumor and metastatic sites by the presence of CXCL12, which is able to shape monocyte polarization toward a protumor M2-like phenotype [36, 39–41].

## 5. Conclusions

In conclusion, although further studies are required to validate the molecular mechanisms and the association between the expression of these EMT- and immune-related markers and BC progression, our findings suggest that the upregulation of the CXCR4/CXCL12 axis and the presence of protumor macrophages in the primary tumor and SLNs sustain the aggressiveness of luminal B BC cells, favoring the generation of a permissive tumor microenvironment and leading to metastatic spread.

## Figures and Tables

**Table 1:** Genes differentially expressed in the OSNA ++ sentinel lymph nodes of relapsing and nonrelapsing luminal B BC patients.

Gene	Fold change	*p* value	Adjusted *p* value^∗^
*TGFB2*	5.2	7.81*E* − 05	3.28*E* − 03
*CD163*	5.5	3.30*E* − 04	6.92*E* − 03
*COL1A2*	8.3	3.40*E* − 03	3.28*E* − 02
*CXCR4*	3.4	4.17*E* − 03	3.28*E* − 02
*ITGAV*	2.9	4.25*E* − 03	3.28*E* − 02
*TWIST1*	5.2	4.69*E* − 03	3.28*E* − 02
*CALD1*	4.1	6.44*E* − 03	3.59*E* − 02
*SNAI2*	3.7	6.84*E* − 03	3.59*E* − 02
*MKI67*	1.9	1.26*E* − 02	5.24*E* − 02
*BMP1*	0.4	1.27*E* − 02	5.24*E* − 02
*LAG3*	3.2	1.40*E* − 02	5.24*E* − 02
*CDH2*	2.5	1.50*E* − 02	5.24*E* − 02
*IL2*	3.4	1.78*E* − 02	5.68*E* − 02
*STEAP1*	5.8	1.89*E* − 02	5.68*E* − 02
*ZEB1*	2.8	2.77*E* − 02	7.76*E* − 02
*FOXC2*	7.6	3.32*E* − 02	8.73*E* − 02
*PGR*	4.3	3.71*E* − 02	9.16*E* − 02
*CDH1*	2.2	5.20*E* − 02	1.21*E* − 01
*VCAN*	2.3	5.53*E* − 02	1.22*E* − 01
*WNT5A*	2.0	7.42*E* − 02	1.49*E* − 01
*HPRT1*	1.8	7.46*E* − 02	1.49*E* − 01
*CD28*	2.2	9.66*E* − 02	1.84*E* − 01
*TMEFF1*	2.7	1.15*E* − 01	2.09*E* − 01
*GSC*	2.7	1.33*E* − 01	2.33*E* − 01
*SNAI1*	2.6	1.53*E* − 01	2.57*E* − 01
*OSM*	2.7	1.67*E* − 01	2.69*E* − 01
*IL10*	2.3	1.79*E* − 01	2.78*E* − 01
*FGF1*	2.2	1.90*E* − 01	2.80*E* − 01
*CSF1*	1.9	1.93*E* − 01	2.80*E* − 01
*AHNAK*	0.8	2.30*E* − 01	3.21*E* − 01
*SOX10*	2.5	2.54*E* − 01	3.43*E* − 01
*TIMP1*	1.4	3.11*E* − 01	4.08*E* − 01
*ESR1*	1.5	4.31*E* − 01	5.48*E* − 01
*VIM*	1.2	4.58*E* − 01	5.66*E* − 01
*IGF1R*	1.3	5.06*E* − 01	6.07*E* − 01
*TGFB1*	0.8	5.37*E* − 01	6.27*E* − 01
*SPARC*	0.9	5.99*E* − 01	6.80*E* − 01
*IGFBP4*	1.1	6.57*E* − 01	7.26*E* − 01
*ERBB2*	1.2	7.07*E* − 01	7.61*E* − 01
*CD4*	1.1	8.42*E* − 01	8.69*E* − 01
*AXL*	1.1	8.48*E* − 01	8.69*E* − 01
*MRC1*	1.0	9.00*E* − 01	9.00*E* − 01

^∗^Benjamini-Hochberg-adjusted *p* values.

## Supplementary Materials

Supplementary Materials Supplementary Table 1: clinical and pathological features of patients enclosed in the discovery cohort (*n* = 40). Supplementary Table 2: clinical and pathological features of patients enclosed in the validation cohort (*n* = 150). Supplementary Table 3: detailed list of the investigated genes. Supplementary Table 4: clinical and pathological features of patients enclosed in the Metabric cohort.
