# Supplementary material for: CXCR4/CXCL12 Signaling and Protumor Macrophages in Primary Tumors and Sentinel Lymph Nodes Are Involved in Luminal B Breast Cancer Progression
Source: Dis Markers. 2018 Apr 16;2018:5018671. doi: 10.1155/2018/5018671 (PMC5926522; doi:10.1155/2018/5018671)
Supplement: Supplementary Materials — Supplementary Table 1: clinical and pathological features of patients enclosed in the discovery cohort (n = 40). Supplementary Table 2: clinical and pathological features of patients enclosed in the validation cohort (n = 150). Supplementary Table 3: detailed list of the investigated genes. Supplementary Table 4: clinical and pathological features of patients enclosed in the Metabric cohort. [file 5018671.f1.pdf]

Supplementary Table 1: Clinical and pathological features of patients enclosed in the discovery cohort (n = 40)

Case Number	Larger lesion size (mm)	CK19 copies	ER (%)	PgR (%)	Ki67 (%)	HER2	Site of relapse
1	25	970000	90	80	30	-	LN, Bone
2	22	32000	95	50	50	-	Liver, Bone, Lung
3	46	15300	95	3	30	-	Breast, Bone
4	29	6800000	95	0	60	-	Bone, Liver
5	17	1400000	85	90	35	-	Breast
6	35	85000	100	100	20	-	Bone, Lung
7	18	45000	100	20	40	-	Liver, Bone
8	23	3060000	90	80	40	-	Liver, Bone
9	13	4050000	100	10	30	-	Lung, Pleura
10	17	4250000	95	0	20	-	Brain
11	12	65000	100	100	20	-	Bone
12	40	3700000	100	90	20	-	Liver
13	50	4900000	100	5	20	-	Bone
14	14	650000	100	80	15	-	Lung
15	9	3700000	80	80	20	-	Lung
16	20	290000	95	50	50	-	Liver
17	22	350000	80	80	60	-	Bone, Liver
18	46	176400	85	50	80	-	Lung
19	29	2400000	95	10	60	-	Breast
20	55	4000000	95	90	35	-	Liver
21	18	75000	100	80	50	-	-
22	19	240000	90	20	35	-	-
23	18	130000	100	40	30	-	-
24	17	220000	85	3	20	-	-
25	26	190000	95	60	60	-	-
26	18	13000	90	60	65	-	-
27	30	34000	95	95	30	-	-
28	46	5600	95	5	50	-	-
29	22	93500	95	2	10	-	-
30	15	17400	98	98	25	-	-
31	18	390000	95	0	50	-	-
32	26	290000	95	30	40	-	-
33	16	5100000	90	7	25	-	-
34	16	100000	90	80	33	-	-
35	16	21400	90	90	24	-	-
36	16	12000	90	85	38	-	-
37	26	41000	90	80	35	-	-
38	26	13000	70	7	35	-	-
39	16	72000	90	90	22	-	-
40	12	290000	90	85	25	-	-

CK19 cytokeratin 19, *ER* estrogen receptor, *PgR* progesterone receptor, *HER2* human epidermal growth factor receptor 2, *LN l*ymph node

Supplementary Table 2: Clinical and pathological features of patients enclosed in the validation cohort (*n* = 150)

Case Number	Tumor size (mm)	Grade	LVI	ER (%)	PgR (%)	Ki67 (%)	HER2	Site of relapse
1	-	3	No	70	0	20	-	Lung
2	-	2	Yes	90	60	20	-	Bone
3	6	2	Yes	90	20	45	-	Bone
4	23	3	Yes	100	70	20	-	LN
5	28	3	No	90	90	30	-	Liver
6	-	3	Yes	70	0	40	-	LN
7	23	3	Yes	90	80	40	-	LN
8	6	3	Yes	90	0	20	-	LN, Bone
9	-	2	Yes	90	3	25	-	Lung
10	-	2	-	90	35	30	-	LN
11	-	2	Yes	90	80	40	-	LN, Bone
12	-	2	Yes	95	90	20	-	LN
13	-	3	Yes	95	40	30	-	LN, Skin
14	-	2	Yes	95	90	20	-	LN, Bone
15	13	2	No	95	20	25	-	Bone
16	-	2	No	90	60	20	-	Skin
17	-	3	Yes	60	0	20	-	Bone, Brain
18	-	3	No	90	30	30	-	Liver, LN
19	-	3	Yes	95	95	20	-	Lung, LN
20	22	3	Yes	90	90	30	-	Liver, Bone
21	-	3	Yes	90	90	20	-	Lung
22	13	3	-	95	75	30	-	Bone
23	-	3	Yes	95	40	30	-	Bone, Lung
24	14	2	-	90	90	20	-	Bone
25	-	-	No	70	5	25	-	Bone, Liver
26	31	3	No	60	60	20	-	Bone
27	22	2	No	100	80	20	-	LN
28	22	3	-	90	95	20	-	-
29	13	3	Yes	95	95	25	-	-
30	-	3	No	90	50	20	-	-
31	-	3	No	70	90	30	-	-
32	4	2	Yes	20	80	30	-	-
33	-	2	Yes	90	50	20	-	-
34	-	2	No	70	90	20	-	-
35	-	2	Yes	40	65	45	-	-
36	-	2	No	100	50	20	-	-
37	-	2	No	90	90	30	-	-
38	-	3	Yes	95	80	20	-	-
39	-	3	Yes	20	1	20	-	-
40	-	2	Yes	90	80	40	-	-
41	11	3	No	90	30	60	-	-
42	-	2	No	95	95	20	-	-
43	-	3	No	95	30	20	-	-
44	-	3	-	10	0	20	-	-
45	28	2	Yes	50	0	30	-	-
46	-	2	No	70	30	20	-	-
47	-	3	-	90	15	20	-	-
48	28	3	Yes	95	95	25	-	-
49	-	3	No	90	55	30	-	-
50	-	3	Yes	45	20	50	-	-

51	18	3	No	95	80	25	-	-
52	-	2	-	95	15	25	-	-
53	25	3	Yes	90	70	40	-	-
54	-	3	Yes	95	95	40	-	-
55	-	2	No	95	95	20	-	-
56	27	2	Yes	90	30	30	-	-
57	-	2	No	95	90	20	-	-
58	25	3	-	70	80	25	-	-
59	-	2	No	90	70	30	-	-
60	-	3	No	90	70	25	-	-
61	-	3	Yes	95	90	30	-	-
62	11	2	No	80	80	25	-	-
63	-	2	No	95	0	40	-	-
64	-	2	No	95	50	25	-	-
65	-	2	No	95	95	20	-	-
66	15	3	No	95	2	40	-	-
67	-	3	No	70	90	20	-	-
68	19	2	No	80	80	70	-	-
69	22	2	-	90	10	35	-	-
70	-	3	No	90	50	30	-	-
71	23	-	Yes	70	0	25	-	-
72	-	3	-	80	70	60	-	-
73	21	1	No	90	90	25	-	-
74	-	2	No	95	95	20	-	-
75	-	3	Yes	75	65	60	-	-
76	7	3	-	40	80	40	-	-
77	-	3	Yes	40	2	40	-	-
78	2	3	No	95	95	30	-	-
79	-	3	Yes	70	5	30	-	-
80	-	2	No	90	0	40	-	-
81	28	2	No	70	90	25	-	-
82	-	1	-	95	80	55	-	-
83	-	2	Yes	95	95	25	-	-
84	-	3	Yes	95	5	50	-	-
85	13	2	No	90	95	20	-	-
86	41	2	Yes	80	60	30	-	-
87	-	3	No	30	60	80	-	-
88	-	2	Yes	95	30	30	-	-
89	-	2	No	100	0	20	-	-
90	13	2	No	90	95	25	-	-
91	-	2	Yes	70	5	30	-	-
92	-	3	No	65	70	55	-	-
93	-	1	No	95	45	50	-	-
94	-	2	No	95	95	20	-	-
95	-	2	No	95	90	20	-	-
96	-	-	No	90	60	40	-	-
97	23	3	No	95	10	20	-	-
98	-	3	No	90	95	20	-	-
99	-	2	No	90	95	20	-	-
100	9	2	-	60	60	30	-	-
101	-	3	No	95	1	30	-	-
102	18	3	No	80	25	70	-	-
103	-	2	No	95	95	20	-	-
104	-	3	No	80	70	45	-	-
105	22	3	Yes	80	80	25	-	-

106	14	3	No	50	60	50	-	-
107	-	2	Yes	70	20	20	-	-
108	-	1	No	95	95	20	-	-
109	-	2	No	90	70	25	-	-
110	-	2	Yes	95	5	30	-	-
111	3	3	No	80	5	25	-	-
112	75	2	Yes	95	25	20	-	-
113	-	2	No	95	60	20	-	-
114	34	3	Yes	90	90	50	-	-
115	26	2	Yes	90	60	65	-	-
116	13	2	Yes	95	95	40	-	-
117	15	3	Yes	80	75	20	-	-
118	-	3	Yes	95	90	85	-	-
119	17	3	No	95	80	60	-	-
120	-	2	Yes	90	80	30	-	-
121	32	2	No	95	95	30	-	-
122	11	3	Yes	90	80	25	-	-
123	15	-	Yes	80	65	30	-	-
124	18	3	No	80	80	40	-	-
125	19	3	Yes	95	50	70	-	-
126	-	2	Yes	95	95	22	-	-
127	23	2	No	95	85	20	-	-
128	-	3	Yes	70	80	70	-	-
129	-	2	No	80	60	20	-	-
130	12	3	No	95	90	25	-	-
131	18	3	No	95	5	40	-	-
132	2	2	No	95	15	20	-	-
133	11	2	Yes	80	70	40	-	-
134	11	3	No	70	5	30	-	-
135	12	2	-	95	80	20	-	-
136	35	3	No	100	40	50	-	-
137	11	2	-	95	40	25	-	-
138	22	3	No	70	70	20	-	-
139	1	2	No	90	90	25	-	-
140	15	2	No	95	65	25	-	-
141	-	3	No	90	90	60	-	-
142	25	2	-	98	95	25	-	-
143	11	2	No	95	95	25	-	-
144	7	2	No	95	100	30	-	-
145	12	2	No	95	90	35	-	-
146	32	3	No	80	50	25	-	-
147	15	3	-	70	40	30	-	-
148	1	2	-	95	95	25	-	-
149	6	2	No	90	50	20	-	-
150	6	3	No	90	70	25	-	-

LVI lymphovascular invasion, *ER* estrogen receptor, *PgR* progesterone receptor, *HER2* human epidermal growth factor receptor 2, *LN* lymph node

Supplementary Table 3: Detailed list of the investigated genes

Gene	Gene Name	Localization
AHNAK	AHNAK nucleoprotein	11q12.2
AXL	AXL receptor tyrosine kinase	19q13.1
BMP1	Bone Morphogenetic Protein 1	8p21.3
CALD1	Caldesmon 1	7q33
CD163	CD163 molecule	12p13.3
CD28	CD28 molecule	2q33
CD4	CD4 molecule	12p13.31
CDH1	Cadherin type 1	16q22.1
CDH2	Cadherin 2, type 1, N-cadherin	18q11.2
COL1A2	Collagen type 1, alpha 2	17q21.33
CSF1	Colony Stimulating Factor 1	1p13.3
CXCR4	Chemokine (C-X-C motif) Receptor 4	2q21
ERBB2	Erb-b2 receptor tyrosine kinase 2	17q12
ESR1	Estrogen Receptor 1	6q25.1
FGF1	Fibroblast growth factor 1	5q31
FOXC2	Forkhead box C2	16q24.1
GSC	Goosecoid homeobox	14q32.1
IGF1R	Insulin-like Growth Factor 1 Receptor	12q23.2
IGFBP4	Insulin-like Growth Factor Binding Protein 4	17q21.2
IL10	Interleukin 10	1q31-q32
IL2	Interleukin 2	4q26-q27
ITGAV	Integrin Alpha V	2q31-q32
LAG3	Lymphocyte Activation Gene 3	12p13.32
MKI67	Marker of proliferation Ki-67	10q26.2
MRC1	Mannose Receptor type C 1	10p12.33
OSM	Oncostatin M	22q12.2
PGR	Progesterone Receptor	11q22-q23
SNAI1	Snail family zinc finger 1	20q13.2
SNAI2	Snail family zinc finger 2	8q11
SNAI3	Snail family zinc finger 3	16q24.3
SOX10	SRY box 10	22q13.1
SPARC	Secreted Protein, Acidic, Cysteine-Rich (osteonectin)	5q31.3-q32
STEAP1	Six Transmembrane Epithelial Antigen of the Prostate 1	7q21
TGF-β1	Transforming Growth Factor Beta 1	19q13.1
TGF-β2	Transforming Growth Factor Beta 2	1q41
TIMP1	TIMP metallopeptidase inhibitor 1	Xp11.3-p11.23
TMEFF1	Transmembrane protein with EGF-like and two follistatin-like domains 1	9q31
TWIST1	Twist family bHLH transcription factor 1	7p21.2
VCAN	Versican	5q14.3
VIM	Vimentin	10p13
WNT5A	Wingless-type MMTV integration site family member 5A	3p21-p14
ZEB1	Zinc finger E-box Binding homeobox 1	10p11.2

Supplementary Table 4: Clinical and pathological features of patients enclosed in the Metabric cohort

Characteristics	Luminal B
**Patients (*n*)**	315
**Mean age: years (IQR)**	64.5 (56.4 – 74.1)
**Mean tumor size: mm (IQR)**	25.0 (18.0 – 31.0)
**Lymph node status**	
Negative (%)	160 (50.8)
Positive (%)	155 (49.2)
**Tumor grade**	
G1-2 (%)	125 (39.7)
G3 (%)	181 (57.5)
Unknown (%)	9 (2.8)
**Tumor stage**	
I-II (%)	254 (80.6)
III-IV (%)	28 (8.9)
Unknown (%)	33 (10.5)

IQR interquartile range, *N* number
